# Correction: Perinatal iron restriction is associated with changes in neonatal cardiac function and structure in a sex-dependent manner

**DOI:** 10.1042/CS-2023-0594_COR

**Published:** 2024-10-16

**Keywords:** anemia, hypoxia, nutrition

The authors of the original article “Perinatal iron restriction is associated with changes in neonatal cardiac function and structure in a sex-dependent manner” 10.1042/CS20230594 would like to correct their paper. In the “Quantitative shotgun proteomics” paragraph of the Methods section, the reference for proteomic data deposited into ProteomeXchange via the PRIDE database was incorrectly listed as PXD028922. The correct reference is PXD043588. The authors apologise for this error and any inconvenience it may have caused.

